# Comparison of Postoperative Analgesic Efficacy of Ultrasound-Guided Bilateral Rectus Sheath Block With That of Local Anaesthetic Infiltration in Patients Undergoing Emergency Midline Laparotomy Surgeries: A Randomised Controlled Trial

**DOI:** 10.7759/cureus.31033

**Published:** 2022-11-02

**Authors:** Akshay LaguduvaH, Srinivasan Swaminathan, M.V.S Satya Prakash, Meenupriya A

**Affiliations:** 1 Anesthesia and Critical Care, Jawaharlal Institute of Postgraduate Medical Education and Research, Puducherry, IND

**Keywords:** abdominal surgery, emergency, wound infiltration, rectus sheath block, ultrasound-guided

## Abstract

Purpose

Rectus sheath block (RSB) is increasingly utilised as a part of multimodal analgesia in laparotomy surgeries. We proposed this study to compare the analgesic efficacy of ultrasound-guided bilateral RSB with local anaesthetic (LA) infiltration. The primary outcome was the visual analogue scale (VAS) at rest and cough. The secondary outcomes were the postoperative morphine consumption, time to first rescue analgesia, incidence of postoperative nausea and vomiting (PONV) and patient satisfaction score.

Methods

In our prospective, single-centre, randomised clinical trial, we enrolled a total of 100 patients undergoing emergency midline laparotomy surgeries. They were randomly allocated into two groups and were administered either LA infiltration (group L, n=50) or ultrasound-guided bilateral RSB (group R, n=50) with 15-20 ml of 0.25% bupivacaine end operatively. The categorical and ordinal variables were analysed using Chi-square/ Fisher’s exact test. The continuous and discrete variables were analysed using Mann-Whitney/independent Student t-test.

Results

The median VAS scores in the postoperative period were significantly lower with RSB when compared with LA. Statistically significant differences in median VAS scores were noticed at one hour (P<0.001), four hours (P=0.001), eight hours (P<0.001), and 12 hours (P=0.014) at rest, and at one hour (P<0.001), four hours (P<0.001) and eight hours (P<0.001) during cough. The median morphine consumption was less with RSB (P<0.001). The time to first rescue analgesia was prolonged with RSB (P<0.001). The incidence of PONV was significantly lower with RSB (P=0.027).

Conclusion

Bilateral ultrasound-guided RSB provides extended postoperative analgesia at rest and cough for patients undergoing emergency laparotomy surgeries when compared with LA infiltration. There was a significant reduction in morphine consumption, incidence of PONV, and prolonged time to first rescue analgesia with RSB.

## Introduction

Unresolved acute postoperative pain from emergency abdominal surgeries may herald prolonged hospitalisation due to pulmonary or cardiac complications. It is also associated with unpleasant sensory experiences from the associated sympathetic stimulation [1-3]. Even though epidural analgesia is used as a favourable modality for postoperative analgesia in abdominal surgeries, it is not feasible to employ the neuraxial technique in patients admitted for emergency abdominal surgeries due to various factors, including hemodynamic instability and coagulopathy. Local wound site infiltration at the end of surgery is the most common technique employed in emergency laparotomies for postoperative analgesia. Rectus sheath block (RSB) has been used as a part of multimodal analgesia, especially when neuraxial techniques are unsuitable [4-6]. However, studies are limited related to the application of this technique in emergency laparotomies. In our study, we compared the analgesic efficacy of ultrasound-guided RSB to local anaesthetic wound infiltration in patients undergoing emergency midline laparotomy surgeries. The primary outcome of our study was the visual analogue scale (VAS) scores at rest and cough in both groups during the postoperative period, and the secondary outcomes were the postoperative morphine consumption, the time to first request for rescue analgesia, the incidence of postoperative nausea and vomiting, and the patient satisfaction of analgesia in both the groups.

## Materials and methods

Study design

After approval from the Departmental Research Committee and Institute Ethical Committee, the study was registered as a prospective, single-centre, observer-blinded randomised clinical trial in the clinical trial registry of India, numbered CTRI/2019/01/017134. The study was conducted from April 2019 to June 2020. Patients between 18 and 70 years of age undergoing emergency midline laparotomy surgeries belonging to the American Society of Anaesthesiologists (ASA) physical status class I to III were included. Patients with bleeding disorders, hepatic or renal impairments, local wound infections, allergy to local anaesthetic (LA), expectant mothers, and those requiring ventilator support and unable to express pain postoperatively were excluded from the study.

Randomisation was done with a computer-generated random number table of varying block sizes. Allocation concealment was done using sequentially numbered, opaque, sealed envelopes (SNOSE). The study subjects were randomised into two groups. In group L, 15 to 20 ml of 0.25% bupivacaine was administered on either side of the midline laparotomy incision after wound closure as LA infiltration. In group R, bilateral RSB under ultrasound (USG) guidance was administered. Bupivacaine solution of concentration 0.25% was prepared by reconstituting 0.5% bupivacaine with normal saline in a 1:1 ratio.

After obtaining informed written consent, the patients were briefed on how to express the visual analogue scale (VAS) of 0-10 cm (0: no pain, 10: worst pain) for reporting postoperative pain and about the utilisation of the patient-controlled analgesia (PCA) device. The patients were shifted inside the operation theatre, and standard monitors, including electrocardiogram (ECG), non-invasive blood pressure (NIBP), and oxygen saturation (SpO_2_), were recorded as a baseline. Intravenous (IV) access was secured, and crystalloids were administered. Patients were pre-oxygenated with 100% oxygen for three minutes. A rapid sequence induction (RSI) was performed using thiopentone and succinylcholine for anaesthetic induction. Fentanyl 2 µg/kg IV was given at induction for analgesia. Patients were intubated, and anaesthesia was maintained with oxygen, air, and an isoflurane mixture. Fifteen minutes before the skin incision, a fixed dose of injection morphine 0.1 mg/kg IV was given for intraoperative analgesia. Muscle relaxation was maintained either with vecuronium or atracurium.

In group L, after the completion of the surgery, 15-20 ml of 0.25% bupivacaine was infiltrated on either side of the midline incision by inserting a 22-gauge (G) needle into the tissue plane 5 mm away from the incision site. The intravascular injection was ruled out by negative aspiration before injecting. A continuous fanning motion technique was used to ensure proper coverage of the incision site.

In group R, bilateral RSB was performed after the completion of the surgery under real-time USG guidance using an in-plane approach. Under sterile precautions, the rectus sheath was identified at its lateral border, and a high-frequency linear probe was placed transversely across the linea semilunaris at or just above the level of the umbilicus. The lateral border of the rectus sheath was identified by the transition from the triple layer of muscle (external oblique, internal oblique, and transversus abdominis) on the lateral side to the single layer of muscle (rectus abdominis) medially. A 22-G needle was inserted, the needle tip was identified in-plane approach, and 15-20 ml of 0.25% bupivacaine was administered in the fascial plane between the rectus abdominis muscle and posterior wall of the rectus sheath, which was confirmed by hydrodissection under USG guidance.

After completion of the analgesic intervention, a reversal agent was administered for neuromuscular recovery, and the patient was extubated and shifted to the recovery room. Post-operatively injection paracetamol 1 g IV was administered eighth hourly to all the patients in both groups. Patients were followed up, and their postoperative pain was assessed by a separate team in the post-anaesthesia care unit (PACU), which was blinded to the mode of analgesia received by the patient. Pain scores were recorded at one, four, eight, 12, and 24 hours after the end of surgery using VAS at rest and during voluntary cough. PCA morphine was initiated for patients appealing for rescue analgesia due to pain. Each actuation delivered 1 mg of morphine with a lockout interval of 10 minutes, and the maximum dose allowed was 10 mg in four hours. The cumulative morphine consumed during the first 24 hours and the time taken to consume the first rescue analgesia were also recorded. The incidence of postoperative nausea and vomiting (PONV) was assessed using a PONV impact scale [7]. The level of patient satisfaction with analgesia was analysed using a Likert scale (1: very dissatisfied, 2: dissatisfied, 3: unsure, 4: satisfied, and 5: very satisfied) [7].

Statistical analysis

The sample size was estimated using the statistical formula for comparing two independent means based on the study by Bashandy and Elkholy [6]. The sample size was estimated to be 50 in each group, with the minimum expected mean difference in pain score between the two groups as 1.8 with a standard deviation of 2.7, 5% level of significance, and 90% power. SPSS software version 19 (IBM Corporation, Armonk, New York) was used to analyze the results statistically. Table and graphical data were developed using Microsoft Excel and Word 2010 (Microsoft Corporation, Hyderabad, India). The distribution of categorical variables such as gender was expressed in terms of frequency (number) and percentage (%) and compared using the Chi-square test/Fisher’s exact test as relevant.

The distribution of continuous and discrete variables such as height, weight, VAS score, cumulative morphine consumption, and time to the first analgesic was expressed in terms of the median with interquartile range (IQR) based on the non-normal distribution of data as estimated by Kolmogorov-Smirnov test of normality. The comparison of these variables was made using the Mann-Whitney test. The continuous variables like age, body mass index (BMI), duration of surgery and length of incision were found to have a normal distribution of data as estimated by the Kolmogorov-Smirnov test and were expressed as mean with standard deviation (SD). The comparison of these continuous variables was performed using the Independent Student t-test. The comparison of ordinal data such as ASA physical status class, episodes of PONV using a PONV impact scale, and level of patient satisfaction using a Likert scale was analysed using the Chi-square test/Fisher’s exact test as relevant. P-value <0.05 was regarded as significant.

## Results

**Figure 1:**
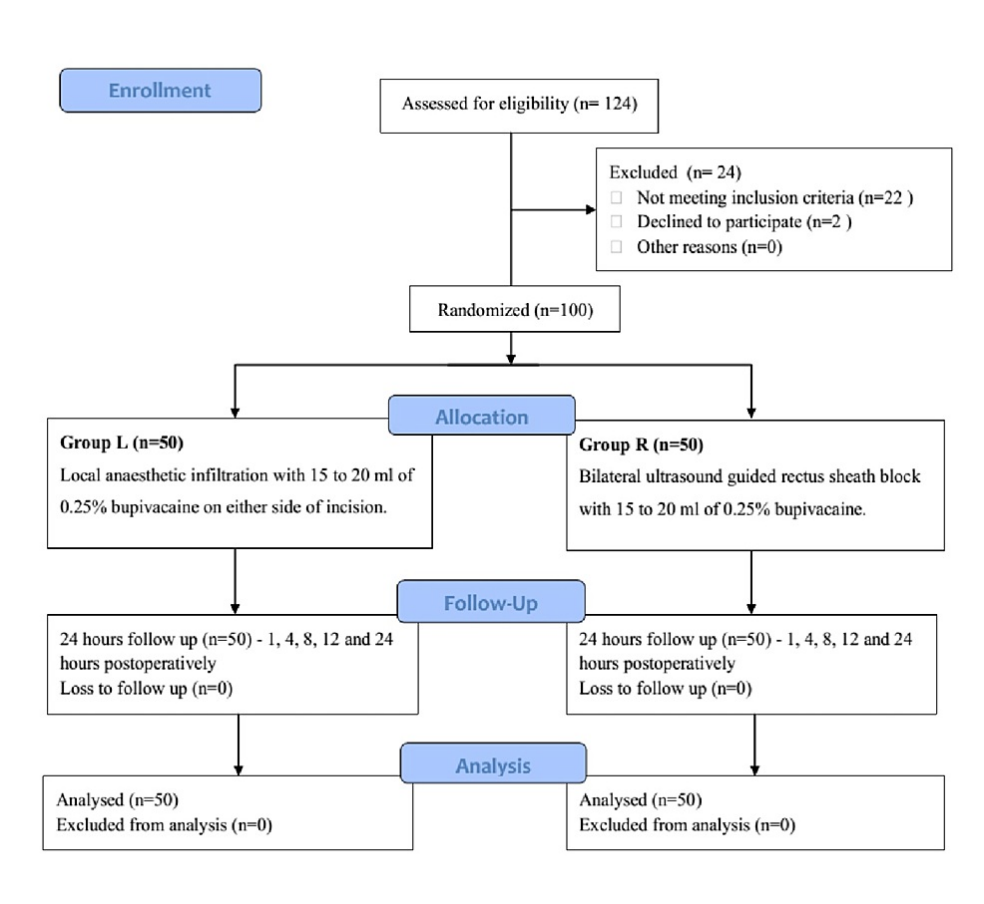
Consort diagram of the trial

A total of 124 patients were enrolled and assessed for eligibility. Out of these, 22 patients were excluded by exclusion criteria, and two patients did not consent to participate. The remaining 100 patients were equally allocated to two groups (Figure 1). The demographic and the baseline perioperative characteristics were comparable in both groups except for weight (P=0.002) and BMI (P=0.003), which were significantly higher in group L than in group R (Table 1). Various types of emergency surgeries, duration of the surgeries, and incision length of the laparotomies are comparable between both groups (Table 2).

**Table 1:** Demographic profile of patients *Statistically significant. ASA PS: American Society of Anaesthesiologists physical status, IQR: interquartile range.

Parameter	Group L (n=50)	Group R (n=50)	P value
Age (years) (mean (SD))	50.4 (13.1)	48.3 (12.8)	0.43
Gender (male/female) (n (%))	40/10 (80/20)	38/12 (76/24)	0.63
Height (m) (median (IQR))	1.6 (1.56-1.65)	1.6 (1.56-1.63)	0.17
Weight (kg) (median (IQR))	60 (55-68)	58 (51.75-60)	0.002*
BMI (kg/m^2^) (mean (SD))	23.7 (2.9)	22.2 (2.1)	0.003*
ASA PS class (I_E_/II_E_/III_E_) (n (%))	0/21/29 (0/42/58)	0/15/35 (0/30/70)	0.21
Duration of surgery (min) (mean (SD))	313.2 (19.9)	319.6 (27.9)	0.19
Length of incision (cm) (mean (SD))	16.6 (0.61)	16.1 (1.52)	0.06

**Table 2:** Surgery type, duration, and incision length in both groups

Type of surgery (n (%))	Group L	Group R	P value
Gastric perforation peritonitis	13 (26)	12 (24)	0.99
Appendicular perforation	14 (28)	16 (32)	
Strangulated umbilical hernia	9 (18)	9 (18)	
Intestinal obstruction	8 (16)	8 (16)	
Blunt trauma abdomen	6 (12)	5 (10)	
Duration of surgery (min) (mean (SD))	313.2 (19.9)	319.6 (27.9)	0.19
Length of incision (cm) (mean (SD))	16.6 (0.61)	16.1 (1.52)	0.06

**Figure 2:**
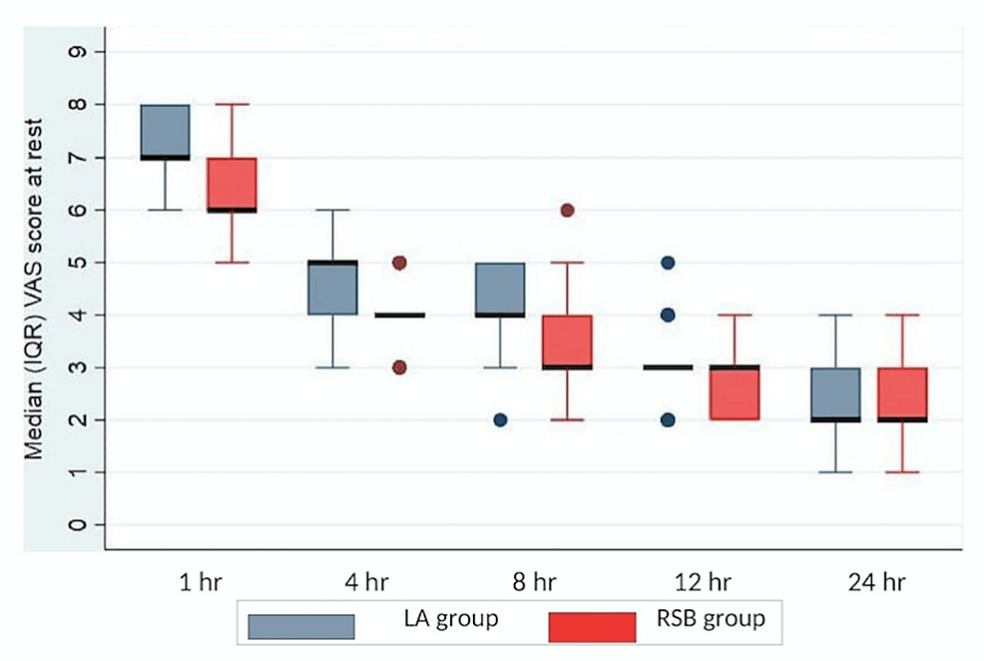
VAS score at rest of both groups in the postoperative period VAS: visual analogue scale, IQR: interquartile range, LA: local anaesthetic, RSB: rectus sheath block.

**Figure 3:**
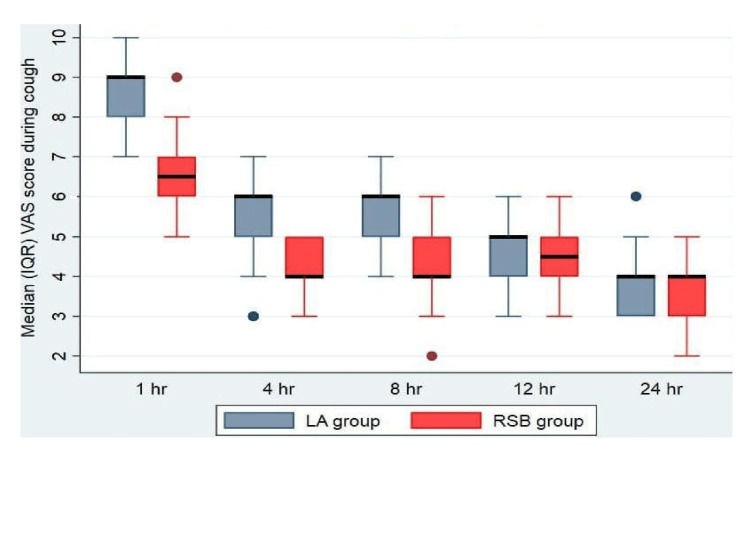
VAS score during cough of both groups in the postoperative period VAS: visual analogue scale, IQR: interquartile range, LA: local anaesthetic, RSB: rectus sheath block.

**Figure 4:**
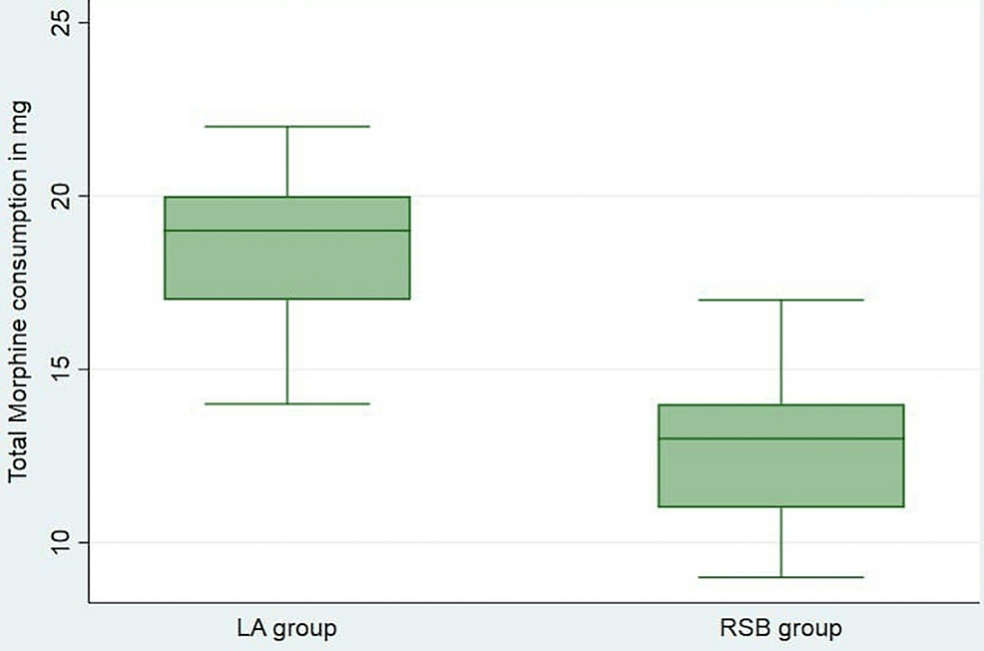
Total morphine requirement in the first 24 hours of postoperative period LA: local anaesthetic, RSB: rectus sheath block.

The median and IQR VAS analysis at rest showed a significantly lesser degree of pain with RSB at one, four, eight, and 12 hours as compared to LA infiltration (Figure 2). The median and IQR VAS analysis during cough showed a significantly lesser degree of pain with RSB at one, four, and eight hours as compared to LA infiltration (Figure 3). However, there was no difference in the VAS between the groups at 24 hours at rest and 12 and 24 hours during cough (Figures 2, 3). The median (IQR) total PCA morphine consumption in group R was 13 (11-14.25) mg and was significantly lower in group L with 19 (17-20) mg, P<0.001 (Figure 4).

**Figure 5:**
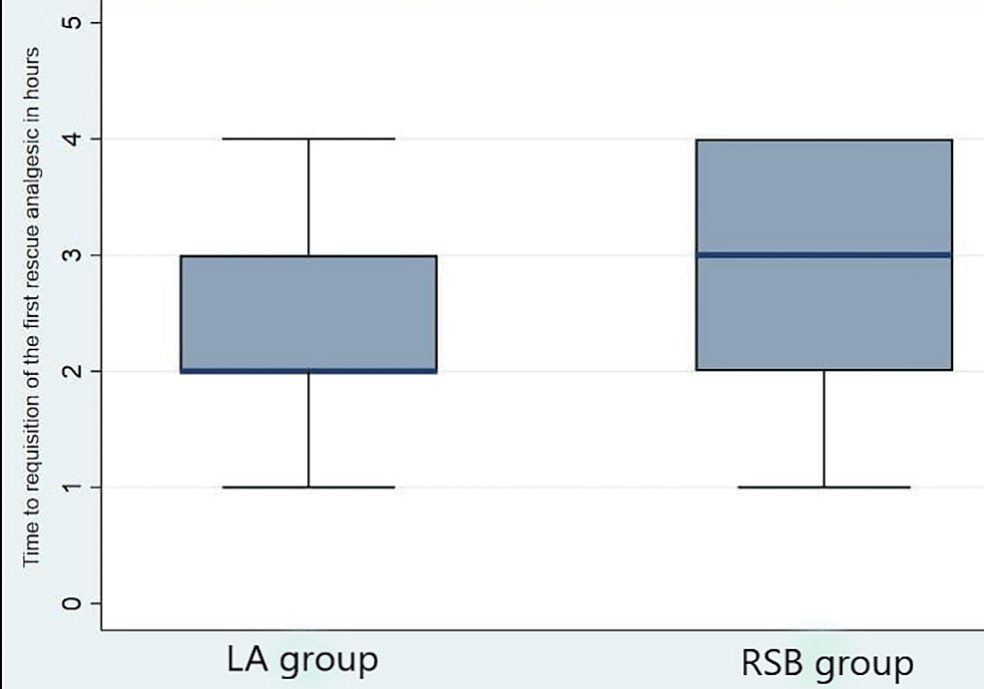
Comparison of the time to requisition of the first rescue analgesic in the postoperative period LA: local anaesthetic, RSB: rectus sheath block.

The median (IQR) time to requisition of the first rescue analgesia was significantly prolonged with RSB, 3 (2-4) hours vs 2 (2-3) hours with LA infiltration, P<0.001 (Figure 5). The incidence of PONV was increased in group L than in group R (P=0.03) (Table 3). When the overall patient satisfaction with analgesia was compared to Likert's scale, we found that a higher number of patients in the RSB group expressed it as “satisfactory” when compared to the LA group (27 patients (54%) in the RSB group vs 18 (36%) in the LA group). The difference between both groups, however, was not statistically significant (Table 4).

**Table 3:** PONV impact score in both groups *Statistically significant. PONV: postoperative nausea and vomiting, RSB: rectus sheath block.

PONV impact score	Group L	Group RSB	P value
0	12 (30)	28 (56)	0.001*
1-3	38 (63.3)	22 (44)	

**Table 4:** Likert's scale for patients' satisfaction with analgesia in both groups

Parameter	Group L (n=50) (n (%))	Group R (n=50) (n (%))	P value
Very dissatisfied	0	0	
Dissatisfied	1	0	0.08
Unsure	31 (62)	21 (42)	
Satisfied	18 (36)	27(54)	
Very satisfied	0	2 (4)	

## Discussion

The findings of our study showed that bilateral rectus sheath blocks given at the end of emergency laparotomy surgeries prolonged the duration of postoperative analgesia compared to local wound infiltration. The postoperative consumption of morphine was also reduced considerably in patients who received rectus sheath block. Regional analgesia techniques have been used widely in midline laparotomy surgeries for the amelioration of surgical stress response and improved patient recovery [8]. Neuraxial techniques like epidural anaesthesia are undesirable in some emergency laparotomy scenarios owing to coexisting hemodynamic perturbations, coagulopathy, and sepsis [4-6]. The use of systemic analgesics like opioids is also limited by their adverse effects [4,5]. Infiltration of LA around the skin incision is one of the traditional methods used, especially when neuraxial techniques are unsuitable [9]. Anterior abdominal wall blocks have gained popularity as a part of multimodal analgesia in recent times. RSB is one of the modalities intended to address the somatic pain from the xiphisternum to the symphysis pubis, innervated by the anterior cutaneous branches of the T7-T12 nerves [10].

In our study, the postoperative median VAS scores were found to be significantly lesser in the bilateral ultrasound-guided RSB group than in the LA infiltration group at one, four, eight, and 12 hours at rest, and at one, four, and eight hours during cough, respectively. There was also a significantly lower median dose of PCA morphine consumption and a prolonged time to first rescue analgesic requirement in the RSB group.

Melesse et al., in a prospective observational cohort study, compared the analgesic effectiveness of bilateral rectus sheath block in patients undergoing emergency midline laparotomy with that of the control group, which was not exposed to any specific intervention. They found that patients undergoing RSB had significantly lower VAS scores at rest and on movement at one, two, four, six, and eight hours but not at 10, 12, and 24 hours points assessed [11]. The analgesic requirement in the first 24 hours was significantly reduced; the time to the first requisition of rescue analgesia was significantly prolonged in the RSB group, and the findings were similar to our study.

Similarly, in a study by Elbahrawy and El-Deeb, the median VAS scores were less at two, four, and six hours postsurgery in the RSB group compared to the control group not receiving any additional intervention [10]. Another study done on patients undergoing laparoscopic surgery by Kasem and Abdelkader found that the patients in the RSB group had lower VAS scores in the period between six to eight hours and eight to 12 hours postoperatively [12].

VAS score is considered a gold standard measure of postoperative pain alleviation [13]. It was used routinely to interpret pain in our institution, and also it was easily understood by most patients. A reduction in VAS score by 1.3 points is considered significant in acute pain [14]. In our study, we found a decline in the median VAS score by 1 and 2 at rest and cough, respectively, in patients who received bilateral RSB (Figures 3, 4). Hence it is evident that RSB can be a promising modality of multimodal analgesia for patients with midline incisions.

The median amount (mg) of PCA morphine required postoperatively was significantly less in the RSB group than in the LA infiltration group (13 (11-14.5) vs. 19 (17-20)), which is in line with the studies done previously by Bashandy and Elkholy, Elbahrawy El-Deeb, and Kasem and Abdelkader [6,10,12]. However, a study by Shah et al. comparing USG-guided RSB with LA infiltration in open hysterectomy or myomectomy surgeries found no difference in the net morphine consumption [15].

The time to first rescue analgesia (in hours) was prolonged significantly in the RSB group. Hence RSB provided extended analgesia compared to LA before supplemental analgesia was needed. This finding was in agreement with the study by Gurnaney et al., which compared RSB to LA infiltration in umbilical hernia surgeries [16]. Kasem and Abdelkader, in their study involving patients undergoing laparoscopic surgeries, found that the time to the first analgesic in the RSB group was comparable to that of the LA infiltration group [12]. This may be due to the visceral pain arising from the irritation of the diaphragm due to the pneumoperitoneum created during laparoscopy. Since the visceral pain could not be relieved by LA infiltration or RSB, these patients would have required opioids in both groups comparably. The incision size for creating laparoscopy ports is usually smaller, and LA could be equally good in reducing analgesic requirements. However, the study by Maloney et al. in laparoscopic appendectomy found a prolonged time to rescue analgesia with RSB, similar to our study [17].

Morphine was administered to all patients to address the visceral pain, according to their requirements, using a PCA pump so that unnecessary administration of IV opioids and its undesirable side effects were prevented. It also allowed an accurate estimation of the amount of morphine consumed. None of the patients experienced any side effects related to morphine except for nausea and vomiting, which was found to be significantly more in the LA infiltration group, which was in agreement with the study by Bashandy and Elkholy [6]. The higher incidence of PONV in the LA infiltration group may be due to the higher requirement of morphine in that group. Ultrasound-guided blocks are performed either as an in-plane or out-of-plane technique. In our study, we chose an in-plane technique as the needle advancement could be visualised throughout its course and would avoid potential complications. Our study had no complications related to the performance of the block, similar to various other studies [17-19].

The study limitation entails the non-observance of the total duration of intensive care unit stay postoperatively and the incidence of postoperative respiratory complications between the groups. Analysis of these data could have better demonstrated the effectiveness of RSB in improving postoperative outcomes. Pain on other sites, like the drainage site, can have a confounding effect on the pain score. Only a single-injection RSB was evaluated in our study. The use of continuous infusion catheters could have improved postoperative analgesia and further reduced the use of opioids in the postoperative period. Sedation and pruritus are more common in patients receiving opioids in the postoperative period, but an analysis of sedation scores and pruritus was not done, which would have shown the benefit of lesser consumption of PCA morphine. The hemodynamic and respiratory parameters were not analysed to assess pain relief as there would be inconsistency in these parameters from other contributing factors in an emergency scenario like fluid loss, anaemia, dehydration, and acidosis.

## Conclusions

Our study showed that bilateral USG-guided RSB provides significantly improved and extended postoperative analgesia at rest and cough for patients undergoing midline laparotomy surgeries on an emergency basis compared to LA infiltration. The significantly reduced requirement of opioids and incidence of PONV in patients who received RSB shows that it can be a promising component of multimodal postoperative analgesia in midline laparotomy surgeries, especially when neuraxial analgesia was not feasible. Further studies can be done by adding adjuvants to LA or using longer-acting LA, which could improve the efficacy of RSB and reduce postoperative complications.
